# Recent Advances on Thermal Management of Flexible Inorganic Electronics

**DOI:** 10.3390/mi11040390

**Published:** 2020-04-09

**Authors:** Yuhang Li, Jiayun Chen, Shuang Zhao, Jizhou Song

**Affiliations:** 1 Institute of Solid Mechanics, Beihang University (BUAA), Beijing 100191, China; liyuhang@buaa.edu.cn (Y.L.); chenjiayun@buaa.edu.cn (J.C.); 2 State Key Laboratory of Structural Analysis for Industrial Equipment, Dalian University of Technology, Dalian 116024, China; 3 Institute of Structure and Environment Engineering, Beijing 100076, China; zhaosh3630@126.com; 4 Department of Engineering Mechanics, Soft Matter Research Center, and Key Laboratory of Soft Machines and Smart Devices of Zhejiang Province, Zhejiang University, Hangzhou 310027, China

**Keywords:** flexible inorganic electronics, thermal management, epidermis, microscale inorganic light-emitting diodes

## Abstract

Flexible inorganic electronic devices (FIEDs) consisting of functional inorganic components on a soft polymer substrate have enabled many novel applications such as epidermal electronics and wearable electronics, which cannot be realized through conventional rigid electronics. The low thermal dissipation capacity of the soft polymer substrate of FIEDs demands proper thermal management to reduce the undesired thermal influences. The biointegrated applications of FIEDs pose even more stringent requirements on thermal management due to the sensitive nature of biological tissues to temperature. In this review, we take microscale inorganic light-emitting diodes (μ-ILEDs) as an example of functional components to summarize the recent advances on thermal management of FIEDs including thermal analysis, thermo-mechanical analysis and thermal designs of FIEDs with and without biological tissues. These results are very helpful to understand the underlying heat transfer mechanism and provide design guidelines to optimize FIEDs in practical applications.

## 1. Introduction

**Figure 1:**
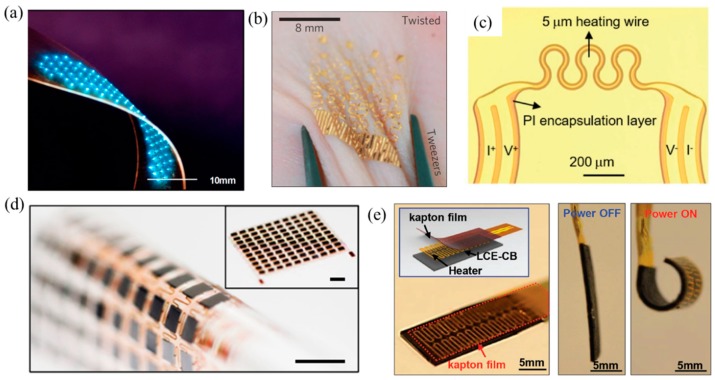
Examples of flexible inorganic electronic devices. (**a**) A flexible inorganic microscale inorganic light-emitting diode (μ-ILED) display [1]. (**b**) An epidermal temperature sensor array in a twisting motion [2]. (**c**) A flexible and stretchable 3ω sensor [3]. (**d**) A soft, thin skin-mounted power management system [4]. (**e**) A soft ultrathin electronics enabled for fully soft robots [5].

Flexible inorganic electronics, which offer the high performance of conventional rigid electronics but with the ability to be deformed like rubber, have enabled many novel devices, with some examples illustrated in Figure 1, including flexible microscale inorganic light-emitting diodes (μ-ILEDs) [1], epidermal electronics [2], flexible and stretchable 3ω sensors [3], skin-mounted power management systems [4], and soft robotics [5]. The challenge to develop flexible inorganic electronic devices (FIEDs) lies in the mismatch between the intrinsic brittle inorganic electronic material (e.g., silicon) and the flexibility requirement in applications. One effective strategy to overcome this mismatch is to integrate the functional inorganic electronic material in a stretchable format [6,7,8,9,10,11,12] at strategic locations on a soft polymer substrate by advanced transfer printing techniques [13,14,15,16]. Due to the unique mechanical properties of FIEDs, they can have conformal contacts with biological tissues under complex deformations, which enable the real-time monitoring of human vital signs for early diagnosis [17]. In the past decade, researchers have demonstrated several flexible real-time health monitoring devices to measure human vital signs including body temperature [4,18], blood glucose [19,20,21], blood oxygen [22], skin hydration [3], blood flow [2], brain electrophysiological signal [23,24], electrocardiography (ECG) [25], etc.

Microscale inorganic light-emitting diodes, as a typical inorganic electronic device, show excellent interactions with biological tissues as excitation sources. For example, μ-ILEDs can be an alternative excitation source to an optoelectronic tweezer via noncontact manipulation methods to investigate behaviors of various biological samples such as cells [26,27,28,29,30,31,32], proteins [33], DNA molecules [34,35] and particles [36,37]. In addition to the above applications, μ-ILEDs can also be adopted in optogenetics to control, affect and readout the neural activities of biological creatures, especially for stimulation in brain [38,39,40,41], retina [42,43,44], and audition [45,46,47,48,49]. However, a large amount of heat energy will be generated by μ-ILEDs when they are in service.

The poor heat dissipation capacity of soft polymer substrate with a thermal conductivity on the order of 0.1 W/m/K in FIEDs, which is much lower than that of the conventional silicon substrate (~100 W/m/K), may induce a high temperature rise in the functional component and cause device performance degradation [50]. When FIEDs are integrated with biological tissues, the sensitive nature of tissues to temperature poses more stringent requirements on the temperature control in practical applications. Moreover, the thermo-mechanical coupling due to the high temperature rise could affect the device reliability [51,52]. Therefore, thermal management of FIEDs becomes one of the most important challenges in the applications of FIEDs, especially in those involving biological tissues. Thermal models will help to establish design guidelines to reduce the adverse thermal effects [53]. This paper aims to review the recent advances in thermal management of FIEDs with the focus on thermal analysis, thermo-mechanical analysis and thermal designs of FIEDs with and without biological tissues, through discussions of analytical models, finite element analysis and experiments. 

## 2. Thermal Management of FIEDs

### 2.1. Thermal Analysis of FIEDs

**Figure 2:**
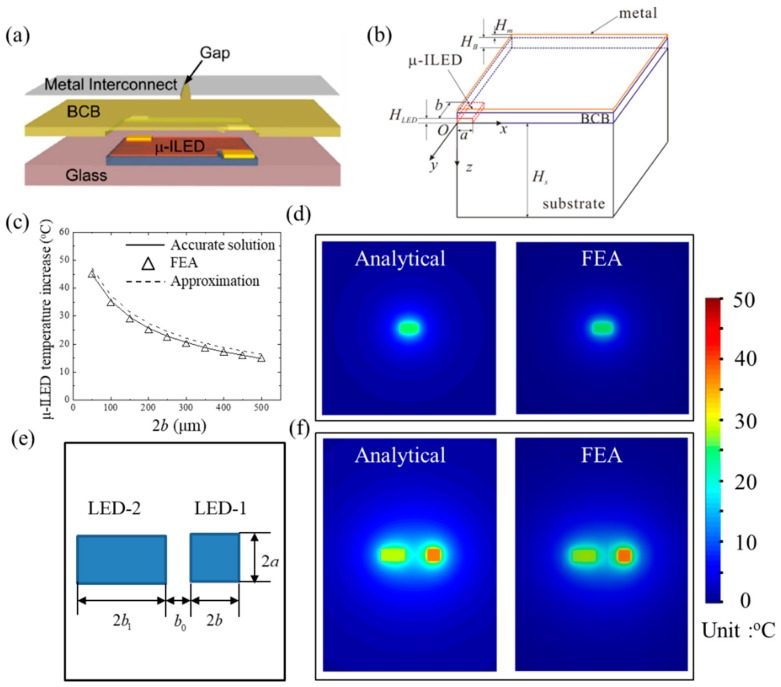
Schematic illustration of (**a**) the μ-ILED layout and (**b**) a quarter geometry of the analytical modeled μ-ILED system [54]. (**c**) The shape influence on the temperature increase of μ-ILEDs with 100 μm × 2*b* × 5 μm under the input power of 12 mW [54]. (**d**) The distribution of the surface temperature increase of a single rectangular μ-ILED under the heat generation power 12 mW [54]. (**e**) Schematic diagram and (**f**) the distribution of surface temperature increase of a μ-ILED array consisting of two μ-ILEDs [54].

We take micro-scale inorganic light-emitting diodes (μ-ILEDs), which serve as the functional components of FIEDs, to induce the undesired heating, as an example to review the recent advances on thermal analysis of FIEDs. Kim et al. [1] reported an unusual strategy using anisotropic etching and microscale device assembly to fabricate μ-ILEDs on unconventional substrates. Figure 2a shows the schematic diagram of the μ-ILED structure with the μ-ILED encapsulated by benzocyclobutene (BCB) and metal layers (i.e., interconnection) on a substrate. Due to the symmetric conditions, a quarter geometry of the μ-ILED layout with key geometrical dimensions labelled is shown in Figure 2b. Cui et al. [54] established a three-dimensional analytical model to study the thermal properties of rectangular μ-ILEDs with the length of 2*a* × 2*b* and a thickness of *H*_LED_. The steady state heat conduction equation can be expressed as

(1) $$\frac{\partial^{2}\Delta T}{\partial x^{2}}+\frac{\partial^{2}\Delta T}{\partial y^{2}}+\frac{\partial^{2}\Delta T}{\partial z^{2}}=0$$

where $\Delta T=T\left(x,y,z\right)-T_{0}$ is the temperature increase from the ambient temperature $T_{0}$. The μ-ILED is modeled as a planar heat source since its thickness is much smaller than its in-plane dimension. The boundary conditions include the heat convection on the top surface, the constant temperature on the bottom surface, and the heat generation in the μ-ILED region. The heat flux and temperature are continuous at the interfaces. The temperature increase of μ-ILED is obtained as

(2) $$\Delta T_{\mathrm{LED}}=\frac{2P}{ab\left[2ab+\left(a+b\right)H_{\mathrm{LED}}\right]\pi^{2}}\int_{0}^{+\infty }\int_{0}^{+\infty }\frac{\left(E+F\right)\sin^{2}\left(\alpha a\right)\sin^{2}\left(\beta b\right)}{G\alpha^{2}\beta^{2}\sqrt{\alpha^{2}+\beta^{2}}}d\alpha d\beta \left(\xi \right)$$

where *P* is the heat generation power; *E*, *F*, *G* are

(3) $$\{\begin{array}{l} E=\left(1+\frac{k_{m}}{k_{B}}\right)\frac{k_{m}\sqrt{\alpha^{2}+\beta^{2}}+h}{k_{m}\sqrt{\alpha^{2}+\beta^{2}}-h}e^{2\left(H_{m}+H_{B}\right)\sqrt{\alpha^{2}+\beta^{2}}}+\left(1-\frac{k_{m}}{k_{B}}\right)e^{2H_{B}\sqrt{\alpha^{2}+\beta^{2}}}\\ F=\left(1-\frac{k_{m}}{k_{B}}\right)\frac{k_{m}\sqrt{\alpha^{2}+\beta^{2}}+h}{k_{m}\sqrt{\alpha^{2}+\beta^{2}}-h}e^{2H_{m}\sqrt{\alpha^{2}+\beta^{2}}}+\left(1+\frac{k_{m}}{k_{B}}\right)\\ G=k_{B}\left(E-F\right)+k_{s}\left(E+F\right)\coth\left(H_{s}\sqrt{\alpha^{2}+\beta^{2}}\right) \end{array}$$

where *k*, *H* and *h* are the thermal conductivity, thickness and coefficient of heat convection, respectively; the subscripts “m”, “*B*”, and “s” stand for interconnection, BCB and substrate, respectively.

To clearly understand the effects of the parameters (such as geometrical parameters, material parameters and loading parameters) on the μ-ILED temperature, Equation (2) was further simplified based on a few reasonable assumptions. First, the effect of the heat convection boundary on the top surface is negligible. Second, the substrate is regarded as a semi-infinite solid because the substrate thickness is much larger than other dimensions. Third, $e^{2H_{m}\sqrt{\alpha^{2}+\beta^{2}}}\approx 2H_{m}\sqrt{\alpha^{2}+\beta^{2}}$, and $e^{2H_{B}\sqrt{\alpha^{2}+\beta^{2}}}\approx 2H_{B}\sqrt{\alpha^{2}+\beta^{2}}$. After adopting the above assumptions, Equation (2) becomes

(4) $$\Delta T_{\mathrm{LED}}\approx \frac{2P}{ab\left[2ab+\left(a+b\right)H_{\mathrm{LED}}\right]\pi^{2}}\int_{0}^{+\infty }\int_{0}^{+\infty }\frac{\frac{\sin^{2}\left(\alpha a\right)\sin^{2}\left(\beta b\right)}{\alpha^{2}\beta^{2}\sqrt{\alpha^{2}+\beta^{2}}}}{\frac{k_{m}H_{m}\sqrt{\alpha^{2}+\beta^{2}}}{1+\frac{k_{m}}{k_{B}}H_{m}H_{B}\left(\alpha^{2}+\beta^{2}\right)}+k_{s}}d\alpha d\beta$$

Figure 2c compares the temperature increase of the μ-ILED with the accurate analytical prediction from Equation (2), the approximate prediction from Equation (4), and finite element analysis (FEA) for μ-ILEDs with 100 μm × 2*b* × 5 μm under the heat generation power of 12 mW. The good agreement among them validates the treatments in analytical models. The temperature increase on the top surface of metal layer is obtained as

(5) $$\begin{array}{l} \Delta T_{\mathrm{surface}}=\frac{4P}{\pi^{2}ab}\cdot \\ \int_{0}^{+\infty }\int_{0}^{+\infty }\frac{\frac{k_{m}H_{m}\sqrt{\alpha^{2}+\beta^{2}}e^{\left(H_{m}+H_{B}\right)\sqrt{\alpha^{2}+\beta^{2}}}}{k_{m}H_{m}\sqrt{\alpha^{2}+\beta^{2}}-h}\sin\left(\alpha a\right)\sin\left(\beta b\right)\sin\left(\alpha x\right)\sin\left(\beta y\right)}{G\alpha \beta \sqrt{\alpha^{2}+\beta^{2}}}d\alpha d\beta \end{array}$$

Again, the good agreement with an error less than 5% of the surface temperature between the analytical prediction from Equation (5) and FEA with power of 12 mW and μ-ILED size of 100 × 200 × 5 μm in Figure 2d validates the analytical model.

The results for a single μ-ILED in Equations (2)–(5) can be adopted to find the temperature of μ-ILED arrays based on the superposition method. Cui et al. [54] took an array of two μ-ILEDs with one’s dimension of 100 × 100 µm (2*a* × 2*b*), the other’s dimension of 100 × 200 µm (2*a* × 2*b*_1_) and the spacing of 200 µm (*b*_0_), as shown in Figure 2e, as an example to illustrate this approach. The temperature increase at any point (*x*, *y*, *z*) for the array of two μ-ILEDs is given by

(6) $$\Delta T_{\mathrm{array}}\left(x,y,z\right)=\Delta T_{1}\left(x,y,z\right)+\Delta T_{2}\left(x,y,z\right)$$

As shown in Figure 2f, the analytical prediction of surface temperature agrees well with FEA, which validates the accuracy of the analytical model. Besides the surface temperature, the temperature increase of μ-ILED array can also be determined with any spacing for an array with finite or even infinite number of μ-ILEDs.

In addition to the 3D thermal model for rectangular heat sources, Lu et al. [55] established an analytical axisymmetric model to study the thermal properties of square μ-ILEDs with an effective radius of $r_{0}=L/\sqrt{\pi }$, with *L* as the length of the μ-ILED, which compares well with FEA and experiments. Yin et al. [56] studied the thermal properties of different shaped serpentine metal heater devices analytically, which can be used to evaluate the heating effects of complex shaped flexible heaters.

**Figure 3:**
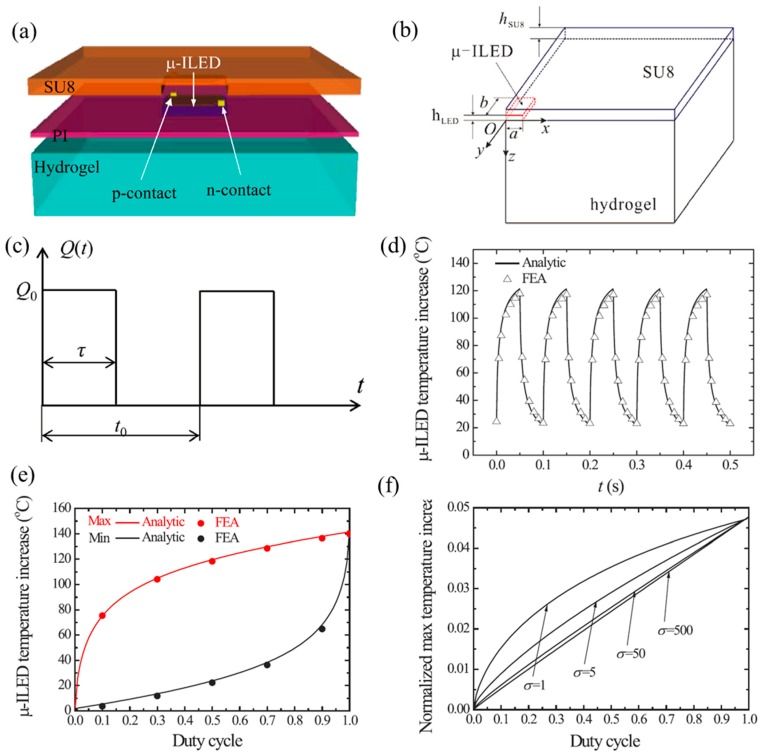
Schematic illustration of (**a**) the μ-ILED layout and (**b**) a quarter geometry of the analytic modeled μ-ILED system [57]. (**c**) The pulsed power *Q*(*t*) with *Q*_0_ as the peak power [57]. (**d**) The μ-ILED temperature increase versus time [57] after saturation [57]. (**e**) The maximum and the minimum temperature increases versus duty cycle after saturation [57]. (**f**) The maximum normalized temperature increase of μ-ILED versus duty cycle with various values of *σ* [57].

Pulsed operation is an effective routine for thermal management by further reducing the adverse thermal influences. Kim et al. [50] fabricated μ-ILEDs, which are located on top of a PI layer and hydrogel substrate and encapsulated with an SU8 layer (Figure 3a) to demonstrate the pulsed strategy. The key geometrical dimensions are labelled in Figure 3b, which shows a quarter geometry of the μ-ILED structure. The applied heat generation power (Figure 3c) is denoted by $Q(t)=Q_{0}U(t)$, where *Q*_0_ is the peak power and *U*(*t*) as a unit pulsed power. Cui et al. [57] established an analytical model to study the thermal properties of μ-ILEDs in pulsed operation. The duty cycle is defined by $D=\tau /t_{0}$, where *τ* is the pulse duration and *t*_0_ is the period.

Figure 3d compares the μ-ILED temperature increase after saturation under pulsed power with the peak power of 30 mW, the period of 50 ms, the duty cycle of 50% and the μ-ILED dimension of 100 × 200 × 6.5 µm (2a × 2b × *h*_LED_) from analytical prediction by Equation (9) and FEA. The good agreement validates the analytical model. In order to study the effect of duty cycle, the maximum and minimum temperature increases of μ-ILED are shown in Figure 3e, which indicates that the duty cycle has a significant effect on the temperature of a μ-ILED. A smaller duty cycle will yield a lower temperature increase of the μ-ILED. Furthermore, Cui et al. [57] established a scaling law for the maximum temperature increase of μ-ILED as

(7) $$\Delta {\bar{T}}_{LED}^{\max}=\frac{{\left(\sqrt{1+\gamma^{2}}\right)}^{3}}{\gamma^{2}\left(1+\frac{1+\gamma }{2\gamma }{\bar{h}}_{LED}\right)}G\left(\chi ,{\bar{h}}_{SU8},D,\gamma ,\sigma \right)$$

where the normalized maximum temperature $\Delta {\bar{T}}_{LED}^{\max}=k_{SU8}a\Delta T_{LED}^{\max}/Q_{0}$, $\chi =k_{hydrogel}c_{hydrogel}\rho_{hydrogel}/\left(k_{SU8}c_{SU8}\rho_{SU8}\right)$, γ=b/a, ${\bar{h}}_{LED}=h_{LED}/a$, ${\bar{h}}_{SU8}=h_{SU8}/a$, $\sigma =a^{2}/\left(\lambda_{SU8}t_{0}\right)$, and *G* is a non-dimensional function [57]. Figure 3f shows the normalized maximum temperature as a function of duty cycle under various values of σ with χ=5.55, γ=2, ${\bar{h}}_{LED}=0.13$, and ${\bar{h}}_{SU8}=0.14$. It is noted that small D or large *σ* are effective to reduce the maximum temperature increase of the μ-ILED. More discussions on the influences of other non-dimensional parameters can be found in Cui et al.’s work [57].

On the other side, many researchers theoretically and experimentally investigated the heat exchange in polymers, which aims to replace metallic heat exchanges for the μ-ILED devices [58,59,60]. A series of new composites were fabricated to enhance the thermal properties of polymers by adding particles [61,62,63], fibres [64,65], carbon nanotubes [66,67,68] and so on.

### 2.2. Thermo-Mechanical Analysis of FIEDs

In addition to effects of temperature increase, the heating induced thermal stress (or strain) is not negligible since the thermal expansion coefficient of sot polymer substrate is much larger than that of the stiff functional inorganic component, which results in noticeable mismatch at the interface between the stiff functional component and the substrate. This mismatch may lead to interfacial delamination, thus causing the failure of FIEDs. Moreover, the non-negligible thermal stress may cause mechanical discomfort when FIEDs are integrated with biological tissues. Therefore, thermo-mechanical analysis of thermal management of FIEDs should be fully explored.

**Figure 4:**
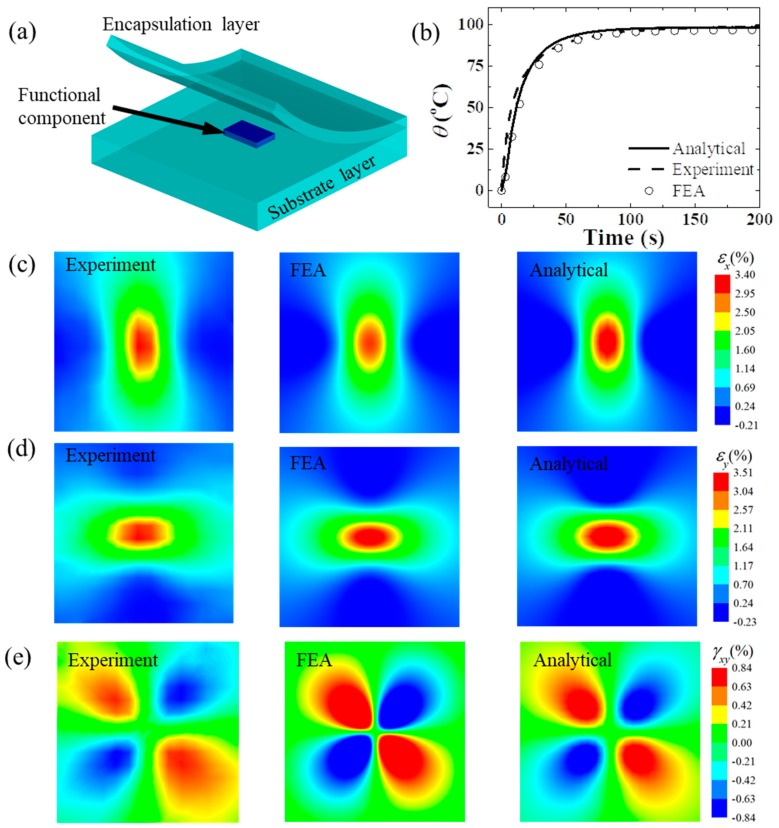
(**a**) Schematic diagram of a flexible electronic device consisting of a functional component on a substrate encapsulated by an encapsulation layer [69]. (**b**) The temperature increase versus time at the center point on the top surface. [69]. The comparisons of (**c**) *x*-stain, (**d**) *y*-strain and (**e**) shear strain from experiments, finite element analysis (FEA) and analytical model [69].

Figure 4a shows a typical structure of an FIED with a functional component encapsulated by an encapsulation layer on a soft polymer substrate. Zhang et al. [69] developed a three-dimensional steady state analytical model to study thermo-mechanical properties of FIEDs based on the transfer matrix method through the Fourier integral transform in space domain and the Laplace integral transform in time domain. According to the linear thermoelasticity theory, the thermo-mechanical equilibrium equations can be expressed as

(8) $$\{\begin{array}{l} \nabla^{2}u+\frac{1}{1-2\mu }\frac{\partial e}{\partial x}-\frac{2\alpha \left(1+\mu \right)}{1-2\mu }\frac{\partial \theta }{\partial x}=0\\ \nabla^{2}v+\frac{1}{1-2\mu }\frac{\partial e}{\partial y}-\frac{2\alpha \left(1+\mu \right)}{1-2\mu }\frac{\partial \theta }{\partial y}=0\\ \nabla^{2}w+\frac{1}{1-2\mu }\frac{\partial e}{\partial z}-\frac{2\alpha \left(1+\mu \right)}{1-2\mu }\frac{\partial \theta }{\partial z}=0\\ \frac{\partial^{2}\theta }{\partial x^{2}}+\frac{\partial^{2}\theta }{\partial y^{2}}+\frac{\partial^{2}\theta }{\partial z^{2}}=\frac{\rho c}{k}\frac{\partial \theta }{\partial t} \end{array}$$

where *θ*, *ρ*, *c*, *t* are temperature increase, density, heat capacity and time, respectively; *u*, *v*, *w* are the displacements along *x*, *y* and *z* directions, respectively; and e=∂u/∂x+∂v/∂y+∂w/∂z is the volumetric strain. After solving the above equilibrium equations combined with boundary and continuous conditions, the stresses, displacements, temperatures and heat fluxes at any point in transformation space are related the counterparts on the bottom surface and can be obtained by

(9) $$\left\{\begin{array}{c} {\tilde{\sigma }}_{z}\left(\xi ,\eta ,z,s\right)\\ {\tilde{\tau }}_{zx}\left(\xi ,\eta ,z,s\right)\\ {\tilde{\tau }}_{yz}\left(\xi ,\eta ,z,s\right)\\ {\tilde{u}}_{z}\left(\xi ,\eta ,z,s\right)\\ {\tilde{v}}_{z}\left(\xi ,\eta ,z,s\right)\\ \tilde{w}\left(\xi ,\eta ,z,s\right)\\ \tilde{\theta }\left(\xi ,\eta ,z,s\right)\\ \tilde{Q}\left(\xi ,\eta ,z,s\right) \end{array}\right\}=\left[\Lambda \right]\left\{\begin{array}{c} {\tilde{\sigma }}_{z}\left(\xi ,\eta ,0,s\right)\\ {\tilde{\tau }}_{zx}\left(\xi ,\eta ,0,s\right)\\ {\tilde{\tau }}_{yz}\left(\xi ,\eta ,0,s\right)\\ {\tilde{u}}_{z}\left(\xi ,\eta ,0,s\right)\\ {\tilde{v}}_{z}\left(\xi ,\eta ,0,s\right)\\ \tilde{w}\left(\xi ,\eta ,0,s\right)\\ \tilde{\theta }\left(\xi ,\eta ,0,s\right)\\ \tilde{Q}\left(\xi ,\eta ,0,s\right) \end{array}\right\}$$

where $\sigma_{z}$, $\tau_{zx}$, $\tau_{yz}$, *Q* are the normal stress, shear stresses and heat flux, respectively; [Λ] is the transfer matrix; the superscript “∼” stands for the variables in the transformation space. The inverse transformation of Equation (9) gives the stresses, displacements and temperature.

In order to validate the analytical model, an experimental setup was established to acquire the strains on the top surface of the device via digital image correlation technology and the temperature via an infrared camera. The FIED with a heater embedded in polydimethylsiloxane (PDMS) is taken as an example to illustrate the analytical approach. Figure 4b compares the temperature increase at the center point on the top surface for a heater (2 × 1.25 mm) under a heat generation power of 291 mW. It is obvious that the temperature rise increases quickly at first and then slowly to the steady state. Figure 4c–e compares the strain contours on the top surface. The good agreement among the analytical predictions, FEA and experiments validates the accuracy of the analytical thermo-mechanical model.

## 3. Thermal Management of FIEDs Integrated with Biological Tissues

### 3.1. Thermal Analysis of FIEDs Integrated with Biological Tissues

The unique mechanical properties of FIEDs, which can sustain complex bending, twisting or stretching even with large deformations, enable their conformal contact with biological tissues for diagnostic and surgical functions. The biological tissues pose great challenges in thermal management. First, the biological tissue is very sensitive to temperature and even a few degrees in temperature increase may cause human discomfort or tissue lesion. Second, the heat transfer in biological tissue doesn’t obey Fourier’s law due to its strong biological features including blood perfusion, metabolism, and so on.

**Figure 5:**
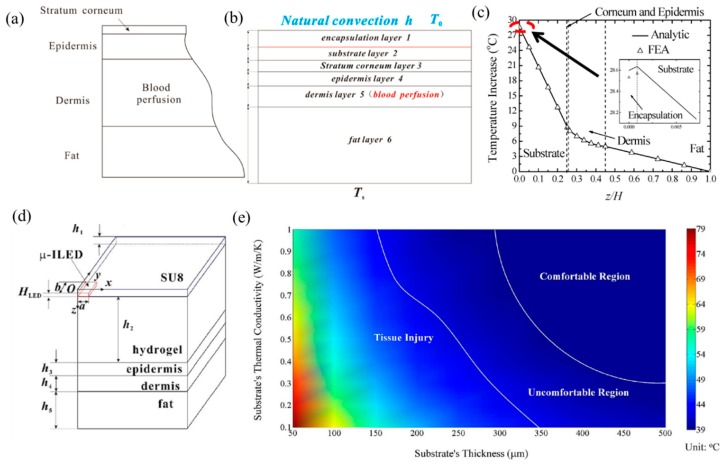
Schematic illustration of (**a**) the skin tissue structure and (**b**) the geometry of the analytical modeled system [70]. (**c**) The comparison of temperature increase along the thickness direction between the analytical prediction and FEA [70]. (**d**) The three-dimensional analytical model showing a quarter geometry of the μ-ILED device integrated with skin tissue [71]. (**e**) The dependence of the thermal comfort map on the thickness and thermal conductivity of the substrate [71].

In the past decade, several analytical studies were performed to study the thermal properties of FIEDs integrated with biological tissues, especially human skin. Cui et al. [70] established a one-dimensional analytical model to predict the temperature in FIED/skin systems. In the model, the skin consists of four layers: stratum corneum layer, epidermis layer, dermis layer and fat layer, as shown in Figure 5a. It is known that blood perfusion only exists in the dermis layer and metabolism exists in all the layers of human skin. The heat transfer equations, including Fourier heat transfer equation in FIEDs and the Pennes bio-heat transfer equation in human skin tissues are given by

(10) $$\{\begin{array}{l} k_{i}\frac{d^{2}\Delta T_{i}}{dz^{2}}=0\;\left(i=1,2\right)\\ k_{i}\frac{d^{2}\Delta T_{i}}{dz^{2}}+q_{\mathrm{met}}=0\;\left(i=3,4,6\right)\\ k_{5}\frac{d^{2}\Delta T_{5}}{dz^{2}}-\omega_{b}\rho_{b}c_{b}\Delta T_{5}+q_{\mathrm{met}}=0 \end{array}$$

where the subscript *i* denotes the corresponding layer, *k* is the thermal conductivity, ΔT is the temperature increase from the ambient temperature; *ω*_b_, *ρ*_b_ and *c*_b_ are the flow rate, density and heat capacity of blood, respectively, and *q*_met_ is the metabolic heat generation. Considering the boundary conditions with natural convection on the top surface and constant temperature on the bottom surface, and continuity conditions (Figure 5b), the temperature increase in the system can be derived analytically by solving Equation (10). Figure 5c shows the temperature increase in the system along the thickness direction. The results from the analytical model agree well with FEA. It is shown that the temperature increase varies nonlinearly in the dermis layer due to the effect of blood perfusion while temperature varies linearly in other layers.

Cui et al. [71] further extended the one-dimensional thermal model to a three-dimensional model to investigate the thermal properties of FIEDs involving a rectangular heating component (e.g., μ-ILED) integrated with human skin. The analytical model accounts for the coupling between the Fourier heat conduction in μ-ILED and the Pennes bio-heat transfer in skin. Figure 5d shows the schematic structure of the FIED/skin system with key dimensions labelled. The governing equations and boundary conditions are all non-homogeneous for such a system. The linear superposition principal is adopted to obtain the temperature increase, which is validated by FEA. Based on the thresholds of 39 and 43 °C for thermal pain and tissue injury, a thermal comfort map is obtained as shown in Figure 5e under the heat generation power of 5 mW, with the comfortable region, uncomfortable region and tissue injury region separated by the two white lines. It is shown that a thicker substrate with a higher thermal conductivity can reduce the maximum temperature increase to avoid human discomfort or tissue injury. Cui et al. [71] also investigated the thermal properties of FIED/skin system under a pulsed heat generation power to further reduce the temperature rise.

In order to consider the actual situations, the clothing effect [72], sweating effect [73], and interfacial thermal resistance effect [74] on thermal properties of the FIED/skin system were also investigated. Besides human skin, thermal properties of FIED integrated with other biological tissues were also studied. For example, Yin et al. [75] developed an axisymmetric cambered analytical model in a sphere coordinate system to investigate the thermal characteristics of FIEDs affixed on a large curvature myocardial surface via steady state analysis. The model, verified by FEA, can exactly predict the thermal properties of the heart or other large curvature structures. Effects of parameters, such as curvature, myocardium, substrate thickness and substrate material on the thermal properties of FIEDs were investigated systematically. Li et al. [76] established an analytical model to predict temperatures of four μ-ILEDs in the brain and yielded optimal designs to constrain the temperature rise below 1 °C [77].

Thermal models of FIED/skin systems can be used to characterize human vital signs and mechanical properties of human skin. For example, Webb et al. [2] developed an ultra-thin, soft, conformal temperature sensor and heater array that can measure blood flow. When the power is applied, the heaters induce an anisotropic temperature distribution, which can give the blood flow based on analytical thermal models. Tian et al. [3] proposed an effective three omega technology to obtain the skin hydration by comparing the analytical model, finite element analysis and experiments for the FIED/skin system under a transient periodical load. Li et al. [78] designed and fabricated flexible sensors on fingers/toenails to precisely study thermal transport characteristics of nail bed tissue.

### 3.2. Thermo-Mechanical Analysis of FIEDs Integrated with Biological Tissues

**Figure 6:**
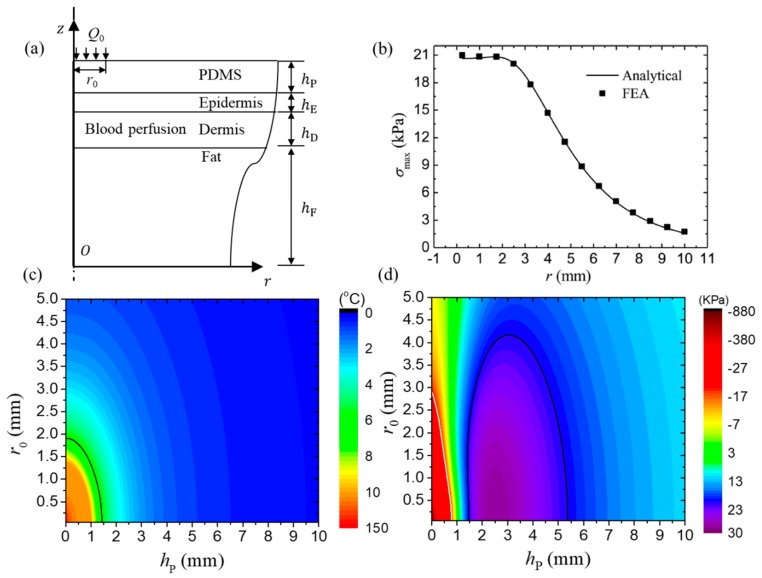
(**a**) Schematic diagram of the cross-sectional structure for the flexible inorganic electronic device (FIED)/skin system [79]. (**b**) The distribution of the maximum principal stress at the device/skin interface along the radial direction [79]. (**c**) The maximum temperature increase and (**d**) the maximum principal stress at the device/skin interface vary with the size of heating component and the substrate thickness [79].

Li et al. [79] established an axisymmetric steady state thermo-mechanical model to obtain the temperature and stresses in the FIED/skin system. Figure 6a schematically shows the multilayer structure of FIED/skin system with the power density of *Q*_0_ loaded in a circular region with radius of *r*_0._ The thicknesses of substrate, epidermis, dermis and fat layers are denoted by *h*_P_, *h*_E_, *h*_D_ and *h*_F_, respectively. The transfer matrix method was adopted to derive the temperature increase and thermal stresses in the system at any point. For example, Figure 6b shows the maximum principal stress distribution at the device/skin interface for a heater size of 2 mm. The analytical predictions agree well with FEA, which validates the accuracy of the analytical thermo-mechanical model. The maximum principal stress reaches an almost unchanged maximum value within the heating region (r < 2 mm) and then decreases quickly as the distance to the heater increases. The distribution of temperature increase at the device/skin interface shows a similar trend as that of the maximum principal stress [79].

The effects of size of the heating component and substrate thickness on the maximum temperature increase and the maximum principal stress are shown in Figure 6c,d, respectively. The black line in Figure 6c gives the critical design parameters, which yield the maximum temperature increase of 6 °C, to separate the thermal comfortable and uncomfortable regions. The region above the black line is the thermal comfortable region while the one below is the thermal uncomfortable region. The black and white lines in Figure 6d give the design parameters, which yield the principal stress with the maximum absolute value of 20 kPa, to separate the mechanical comfortable and uncomfortable regions. The region above the black and white lines is the mechanical comfortable region and the ones below are mechanical uncomfortable regions. These results are very helpful to provide design guidelines to reduce the undesired thermal responses in the FIED/skin system.

To further investigate the heater shape effect on the thermo-mechanical properties of the FIED/skin system, Zhang et al. [80] established a three-dimensional analytical steady state model to study the thermo-mechanical behaviors of the FIED/skin system with rectangular heating components based on the transfer matrix method. The transfer equation for each layer is derived analytically. Both cases of one heater and multiple heaters were systematically investigated.

**Figure 7:**
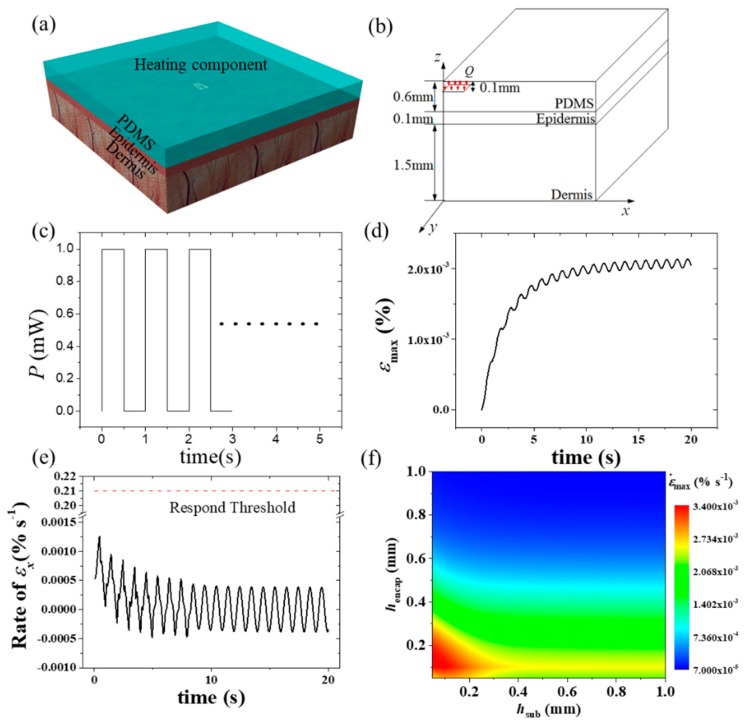
Schematic diagrams of (**a**) the multilayer structure of the device/skin system with one rectangular heating component and (**b**) the analytical modeled system with a quarter of the geometry [81]. (**c**) Periodic pulsed power density versus time [81]. (**d**) Maximum principal strain. (**e**) Maximum rate of principal strain as functions of time in the skin [81]. (**f**) Influences of thicknesses of encapsulation and substrate on the peak values of the maximum rate of the principal strain [81].

Besides the steady state thermo-mechanical analysis, a transient thermo-mechanical model is also important considering that the perceptions of thermoreceptors and mechanoreceptors, as uncomfortable feelings are related to the response of thermoreceptors, the rate of thermal strain, and the thermal stress with thresholds of 19.8 imp/s, 0.21%/s and 20 kPa, respectively [81]. Zhang et al. [81] established a three-dimensional transient analytical model to predict the temperature increase and thermal stresses in the FIED/skin system with the structure layout shown in Figure 7a,b. Both cases, under a constant heat generation power and a pulsed power (Figure 7c), were systematically studied. Figure 7d–f shows some typical results under a pulsed heat generation power for the FIED/skin system with a heating component size of 1 mm × 1 mm and a thickness of PDMS encapsulation of 0.6 mm. The pulsed power density used for calculations has a magnitude of 1mW/mm^2^, pulse duration time of 0.5 s and period of 1 s. The maximum principal strain and strain rate are given in Figure 7d,e, respectively. The thermoreceptors and mechanoreceptors in the human body help humans feel temperature, strain or stress. Once the responses of these receptors exceed the thresholds, humans may have an uncomfortable feeling. The red dotted line in Figure 7e corresponds to the response threshold of strain rate (0.21%). It is noted that the maximum principal strain rate is below 0.21%, which indicates that the strain rate is not large enough to induce mechanical discomfort under these specific loading conditions. Figure 7f shows the influences of thicknesses of encapsulation and substrate on the peak value of the maximum rate of the principal strain. These results are very useful to determine whether the strain rate can induce mechanical discomfort. In addition to the maximum rate of the principal strain, the maximum thermoreceptor response and the maximum principal stress were also systematically investigated to study whether the temperature and thermal stress induce human discomfort in Zhang et al.’s work [81].

## 4. Thermal Designs of FIEDs

In practical applications of FIEDs integrated with biological tissues, it is important to prevent the undesired heating induced by FIEDs to dissipate to biological tissues through the substrate of FIEDs. Several active thermal designs [82,83,84,85,86] have been reported to help thermal management of the FIED/skin system. This section will briefly review the recently developed orthotropic substrate design [83] and the functional soft composite design [85].

### 4.1. Orthotropic Substrate Design

**Figure 8:**
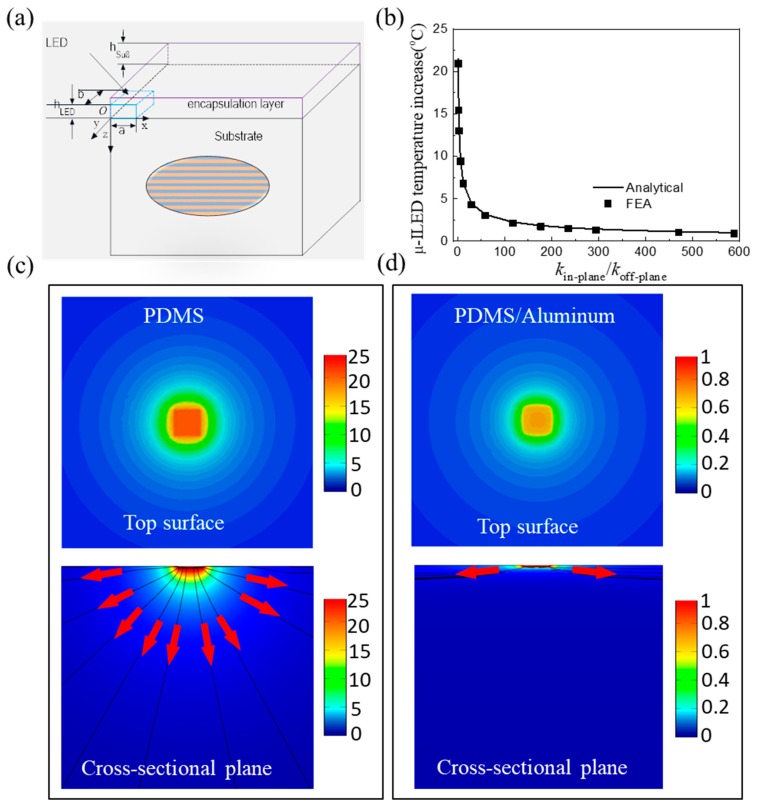
(**a**) Scheme diagram of the μ-ILED device on an orthotropic substrate consisting of two layered materials [83]. (**b**) The temperature increase of the μ-ILED versus the thermal conductivity ratio [83]. Distributions of temperature increase for the μ-ILED device on the (**c**) isotropic and (**d**) orthotropic substrate [83].

In order to prevent heating energy to dissipate from FIEDs to human skin, Li et al. [83] reported an orthotropic substrate design consisting of layered metal and polymer materials shown in Figure 8a, which can control the heat flow direction to achieve the goal of thermal management. The in-plane and off-plane thermal conductivities can be obtained as

(11) $$k_{\mathrm{in}\text{-}\mathrm{plane}}=\frac{k_{1}+k_{2}}{2},\;k_{\mathrm{off}\text{-}\mathrm{plane}}=\frac{2k_{1}k_{2}}{k_{1}+k_{2}}$$

where *k*_1_ and *k*_2_ are the thermal conductivities of metal and polymer materials, respectively. For a large difference in *k*_1_ and *k*_2_, Equation (11) yields $k_{\mathrm{in}\text{-}\mathrm{plane}}\gg k_{\mathrm{off}\text{-}\mathrm{plane}}$, which indicates that the heat dissipation along the in-plane directions is much larger than that along the off-plane direction. This unique orthotropic feature offers an advantage in thermal management of FIEDs, especially in bio-integrated applications where the heat dissipation along the off-plane direction should be minimized.

The μ-ILED is taken as the heating component in FIEDs to demonstrate the performance of orthotropic substrate design and to identify the underlying heat transfer mechanism. Li et al. [83] established a three-dimensional analytical model to study the thermal properties of FIEDs with an orthotropic substrate. Figure 8b shows the influence of the orthotropic substrate design on the temperature of the heating component. The analytical prediction agrees well with FEA. The μ-ILED decreases quickly as the in-plane thermal conductivity of orthotropic substrate increases. For the thermal conductivity ratio $k_{\mathrm{in}\text{-}\mathrm{plane}}/k_{\mathrm{off}\text{-}\mathrm{plane}}$ of 60, the μ-ILED temperature has a significant decrease from 21.5 to 3 °C. Figure 8c,d shows the temperature distributions on the top surface and cross-sectional plane for the cases of homogeneous PDMS substrate and orthotropic PDMS/aluminum substrate, respectively. Indeed, the orthotropic substrate changes the heat dissipation path with much more heat dissipating along the in-plane directions, as indicated by the black lines and red arrows. Li et al. [84] further studied the thermal properties of FIEDs on an orthotropic substrate for biointegrated applications with influences of thermal conductivity of orthotropic substrate, substrate thickness, and loading parameters fully investigated. It was shown that the orthotropic substrate design can reduce the temperature rise of skin significantly.

### 4.2. Functional Soft Composite Design

**Figure 9:**
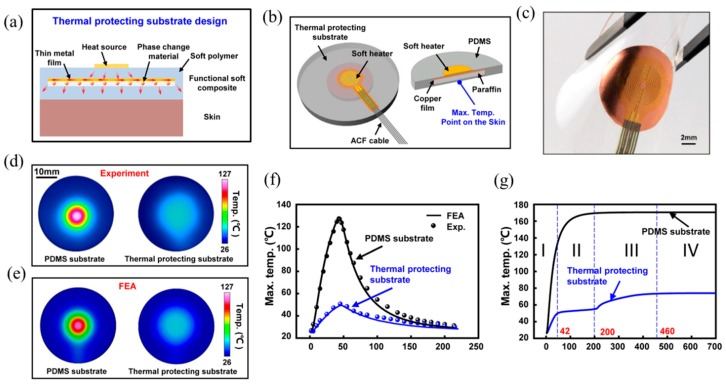
(**a**) Cross-sectional illustration of a skin-integrated wearable device with a functional soft composite substrate design [85]. (**b**) Schematic illustration of a wearable device consisting of a heat source on the functional soft composite substrate with the embedded paraffin as the phase change material and the Cu film on its top in PDMS [85]. (**c**) Optical image of a wearable device under bending deformation [85]. Temperature distributions on the bottom of the PDMS substrate and thermal protecting substrate from (**d**) experiments and (**e**) FEA after heated 40 s [85]. The maximum temperature on the bottom of the thermal protecting substrate versus time with (**f**) a heating duration of 40 s and (**g**) a long heating duration [85].

Shi et al. [85] proposed a functional soft composite to serve as the thermal protecting substrate for wearable electronics, with abilities to manipulate the heat flow and efficiently absorb the excessive heat energy. The thermal protecting substrate features embedded phase change materials with a thin metal film on the top in a soft polymer as shown in Figure 9a. The thin metal film helps to spread the heat energy along in-plane directions while the phase change material helps to absorb the heat energy once the temperature exceeds the transition temperature. A wearable electronics with soft heater as the heating component, copper as the metal film, paraffin as the phase change material and PDMS as the substrate material was developed as shown in Figure 9b. Figure 9c shows the optical image of a wearable device under bending deformation, which indicates its ability to have a conformal contact with skin.

Figure 9d compares the temperature contour on the bottom surface of the substrate between wearable electronic devices on a conventional PDMS substrate and a thermal protecting substrate from experiments, which can obviously show the high efficiency of the functional thermal protecting substrate in thermal management. In order to reveal the underlying physics associated with the thermal protection, a three-dimensional finite element model was established. Figure 9e shows the temperature distributions on the bottom surface of FEA, which agree well with experiments. Figure 9f compares the maximum temperature on the bottom surface with a heating duration time of 40 s. The good agreement between experiments and FEA again validates FEA. Figure 9g shows the temperature versus time to reveal the thermal protection mechanism. For wearable electronic devices with a conventional substrate, the temperature increases monotonically to steady state, while the temperature response can be described as four stages for wearable electronic devices with a thermal protecting substrate. At stage I, the temperature is reduced compared to the case of conventional substrate, mainly due to the heat flow manipulation of Cu film since the temperature is below the transition temperature of paraffin. At stage II, the temperature remains unchanged due to the occurrence of phase change of paraffin from sold state to liquid state and the excessive heat energy is absorbed during the phase change process. At stage III, the temperature increases monotonically to steady state, i.e., stage IV. It is shown that this thermal protecting substrate design can reduce the maximum temperature increase over 85% with optimal parameters.

The soft functional composite design in Figure 9a can only provide limited stretchability due to the existence of a stiff metal film. To further improve the stretchablity, Shi et al. [86] developed a composite with paraffin uniformly mixed with PDMS, which can be bent, twisted and stretched easily without losing the ability to absorb the excessive heat energy.

Other methods were also developed to effectively reduce the temperature through novel designs. For example, Kotagama et al. [87] adopted a thermally conductive composite and included liquid-cooled tubes to enhance the electronic cooling. Liquid metal inclusions were also embedded into the polymer substrate to achieve both flexibility and high thermal conductivity [88,89].

## 5. Conclusions

This paper overviews the recent advances on thermal management of FIEDs including thermal analysis, thermo-mechanical analysis and thermal designs of FIEDs with and without biological tissues to provide design guidelines for optimization. Despite these advances on thermal management of FIEDs, there are still many open challenges and opportunities for future research. For example, for FIEDs with high heat generation powers such as a flexible inorganic μ-ILED display with high resolution, active thermal design with associated thermal modeling to avoid the undesired heating is required. In addition, for FIEDs integrated with biological tissues, the thermal pain sensation based on a holistic neurodynamics model is needed for accurate modeling.
